# Morroniside Ameliorates Endotoxin-Induced Uveitis by Regulating the M1/M2 Polarization Balance of Macrophages

**DOI:** 10.1155/2023/1252873

**Published:** 2023-04-23

**Authors:** Wenjie Li, Lin Liu, Ziwei Zhang, Hong Lu

**Affiliations:** ^1^The First Clinical Medical College, Lanzhou University, Lanzhou 730000, China; ^2^Department of Ophthalmology, The First Hospital of Lanzhou University, Lanzhou 730000, China; ^3^Department of Ophthalmology, Beijing Chao Yang Hospital, Capital Medical University, Beijing 100083, China

## Abstract

**Background:**

Inflammation is closely associated with the pathogenesis of various ocular diseases. Uveitis is a condition characterized by the inflammation of the uvea and ocular tissues that causes extreme pain, decreases visual acuity, and may eventually lead to blindness. The pharmacological functions of morroniside, isolated from *Cornus officinalis*, are multifarious. Morroniside exerts various therapeutic effects, e.g., it ameliorates inflammation. However, the specific anti-inflammatory effect of morroniside on lipopolysaccharide-induced uveitis has not been reported widely. In this study, we investigated the anti-inflammatory effect of morroniside on uveitis in mice.

**Methods:**

An endotoxin-induced uveitis (EIU) mouse model was constructed and treated with morroniside. The inflammatory response was observed using slit lamp microscopy, and histopathological changes were observed by hematoxylin–eosin staining. The cell count in the aqueous humor was measured using a hemocytometer. The concentrations of TNF-*α*, IL-6, and IL-1*β* in the ciliary body and retina were measured using ELISA kits. The expression of iNOS and Arg-1 in the ciliary body and retina was measured by immunofluorescence costaining, and western blotting was performed to measure the protein expression of JAK2, p-JAK2, STAT3, and p-STAT3 in the ciliary body and retina.

**Results:**

Morroniside effectively ameliorated the inflammatory response in EIU mice. Furthermore, morroniside significantly reduced the concentrations of IL-1*β*, IL-6, and TNF-*α* in the ciliary body and retina. Morroniside treatment significantly reduced the expression of iNOS in the ciliary body and retinal tissues. It also significantly inhibited p-JAK2 and p-STAT3 expression and promoted Arg-1 expression. In addition, morroniside boosted the effect of JAK inhibitors on the above indices.

**Conclusions:**

Collectively, these findings suggest that morroniside may protect against LPS-induced inflammation in uveitis by promoting M2 polarization through the inhibition of the JAK/STAT pathway.

## 1. Introduction

Uveitis is a serious inflammatory disease of the eye and a major blinding disease, occurring primarily in young adults. Blindness caused by uveitis is irreversible and mostly bilateral [1]. In China, there are ∼3–4 million patients with uveitis [2]. Uveitis can be classified as anterior, intermediate, posterior, and total uveitis depending on the anatomical location, with anterior uveitis being the most frequent, accounting for 50%–92% of all cases of uveitis [3]. Because the pathogenesis of anterior uveitis is not well understood, no effective treatment has been identified for the condition [4]. Although immunosuppressants and hormones are effective, their clinical effects are unstable, and the condition is prone to recur with these treatments, eventually leading to the loss of visual function or vision [5]. Therefore, the search for novel treatment methods is one of the most important topics in the field of uveitis research.

Morroniside is an iridoid extracted from the traditional Chinese medicine *Cornus officinalis* [6]. Studies have shown that morroniside modulates the downregulation of apoptotic proteins in osteoarthritis to exert antiapoptotic effects [7], promotes angiogenesis after myocardial infarction, and improves the prognosis of myocardial infarction, besides exerting anti-inflammatory effects [8]. In addition, morroniside was shown to exert anti-inflammatory pharmacological effects in cases of aseptic inflammation, including that in cerebral ischemia-reperfusion [9], chronic atrophic gastritis [10], inflammatory cell infiltration in acute colitis [11], and liver and kidney injury induced by type 2 diabetes in mice [12]. However, only a limited number of studies have reported the improvement of uveitis upon morroniside treatment.

Endotoxin-induced uveitis (EIU) is an efficient animal model for the study of pathological mechanisms associated with uveitis [13]. The condition is primarily characterized by signs of acute anterior uveitis, such as ciliary congestion, iris blood vessel dilatation, anterior chamber exudate secretion, pupil occlusion, and fibrous membrane formation. In our previous study, we found that macrophages in the iris and ciliary body were activated in EIU. The morphology of a large number of cells were altered significantly, and TLR4 expression on the surface of macrophages was significantly upregulated [14].

Macrophages are important components of innate immunity and play a central role in inflammation and host defense [15]. Under different pathophysiological conditions, macrophages can be transformed into different functional phenotypes, namely classically activated macrophages (M1) and alternatively activated macrophages (M2) [16]. A decline in the abundance of anti-inflammatory M2 macrophages and an increase in that of proinflammatory M1 macrophages were associated with the development of inflammatory diseases. An imbalanced M1/M2 macrophage ratio is an important pathological factor driving the development of inflammatory diseases [17, 18]. Considering the important role of the M1/M2 macrophage balance in inflammatory diseases, its application in the management of uveitis may be a novel method to control inflammatory damage.

To address the research topic, we used mice with EIU treated or without morroniside. We compared the inflammatory response and histopathological changes and measured the level of inflammatory factors, the extent of macrophage M1/M2 polarization, and changes in the JAK/STAT pathway after treatment. We aimed to investigate the regulatory effect of morroniside on the balance in M1/M2 macrophage polarization in EIU mice to understand the theoretical basis for EIU treatment and develop suitable treatment methods.

## 2. Materials and Methods

### 2.1. Animals and Reagents

Forty-eight BALB/c mice (half male and half female; body mass 22–28 g) were purchased from the Experimental Animal Center of Lanzhou University (No. SCXK (Gan) 2018-0002). The mice were housed at a temperature of (25 ± 1)°C, relative humidity of 65% ± 10%, and a 12 hr light–dark cycle. All studies were conducted in accordance with the recommendations of the Guide for the Care and Use of Laboratory Animals and in compliance with the regulations of the Gansu Provincial Animal Management Committee. All necessary efforts were made to minimize animal suffering and reduce the number of animals used in the experiments.

Lipopolysaccharide and morroniside were purchased from Shanghai Yuanye Bio-Technology Co., Ltd. Anti-iNOS, anti-COX2, anti-Arg-1, anti-CD206, anti-JAK2, anti-p-JAK2, anti-STAT3, anti-p-STAT3, and goat anti-rabbit IgG-HRP antibodies were purchased from Abcam (Cambridge, UK). TNF-*α*, IL-6, and IL-1*β* ELISA kits were purchased from Shanghai ZCIBIO Technology Co., Ltd. PBS and trypan-blue solution were purchased from Beijing Solarbio Technology Co., Ltd. A bicinchoninic acid assay (BCA)-100 Protein Quantitative Analysis Kit was purchased from Shanghai Biocolor Biotechnology Co., Ltd. RIPA and BCA protein assay kits were purchased from Beyotime, Beijing. ECL was purchased from Affinity, Shanghai. AG490 was purchased from Shanghai Zerun Bio.

### 2.2. Animal Model and Experimental Groups

After 1 week of adaptive feeding, the mice were randomly divided into the control group (control, *n* = 12), model group (EIU, *n* = 12), morroniside low-dose group (Mor-L, *n* = 6), morroniside high-dose group (Mor-H, *n* = 6), JAK inhibitor (AG490) treatment group (AG490, *n* = 6), and morroniside high dose + AG490 group (Mor-H + AG490, *n* = 6). Mice in the Mor-L and Mor-H groups were gavaged with 30 and 120 mg/kg Mor, respectively. An equal volume of saline was administered to mice in both the control and EIU groups, and mice in the AG490 group were injected subcutaneously with 1 mg/kg AG490. After 5 days of continuous administration, mice in the EIU group, Mor-L group, Mor-H, AG490, and Mor-H + AG490 group were intravitreally injected with 2 *μ*L of lipopolysaccharide to establish a mouse EIU model.

### 2.3. Slit Lamp Observation and Clinical Evaluation

In each group of mice, slit-lamp microscopy (Keeler, UK) was used to observe the inflammatory response, and clinical signs were imaged and recorded. The severity of ocular inflammation was evaluated according to the scoring criteria (Table 1) [19]. Mice in each group were punctured with a 30-gauge needle in the anterior chamber under the microscope, and the aqueous humor (15–20 *μ*L) was collected from both eyes. The anterior chamber, iris, ciliary body, vitreous cavity, and retina of the mice were isolated, and the anterior chamber, iris, and vitreous cavity samples were fixed with 4% paraformaldehyde for histopathological evaluation. The ciliary body and retina samples were partly fixed in 4% paraformaldehyde for histopathological evaluation, and the residual tissues were stored at −80°C for biochemical assays and western blotting.

### 2.4. Cell Count and Protein Concentration in the Aqueous Humor

For cell counting and the measurement of the protein concentration in the anterior chamber, aqueous humor was collected by anterior chamber puncture 24 hr after LPS injection. The aqueous humor was diluted with an equal volume of trypan blue, following which the number of viable and nonviable cells was counted manually using a hemocytometer. For protein level measurement, the aqueous humor was centrifuged for 5 min at 250 × *g*, and the supernatant was isolated. The protein concentration was measured using the bicinchoninic acid assay (BCA)-100 Protein Quantitative Analysis Kit.

### 2.5. Hematoxylin–Eosin (HE) Staining

Fixed tissues that were paraffinized were sectioned at a thickness of 5 *μ*m. HE staining was performed, and histopathological changes were observed using a fluorescence microscope (Motic China Group Co., Ltd.).

### 2.6. Biochemical Assays

The concentrations of TNF-*α*, IL-6, and IL-1*β* in the iris ciliary and retinal cells were measured using ELISA kits. Experimental procedures were conducted strictly in accordance with the manufacturer's instructions.

### 2.7. Immunofluorescence Costaining

The ciliary body and retinal tissues were fixed, paraffin-embedded, sliced (thickness: 3 *μ*m), and dewaxed. Following this, the tissue sections were washed with PBS three times, treated with protease *K* (added dropwise), and incubated at 37°C for 40 min. Following this, the tissue sections were treated overnight with iNOS and Arg-1 or IBA1 fluorescent antibodies (Servicebio, 1 : 200) at 4°C. Following this, the sections were rinsed with PBS three times. After washing, the nuclei were restained with DAPI. The slices were then treated with an autofluorescence quencher and sealed with glycerol. Negative controls (NC, treatment with PBS instead of fluorescent antibodies) were prepared using the same process. Following this, the sections were observed under a fluorescent microscope and imaged under an Olympus laser scanning confocal microscope.

### 2.8. Western Blotting

Proteins from the ciliary body and retinal tissues were extracted by lysing the tissues with RIPA buffer, and the protein concentration was measured using a BCA protein assay kit. The supernatant was mixed with an SDS-PAGE sample loading buffer and transferred to PVDF membranes, which were incubated at room temperature in 5% skimmed milk for 2 hr. The membranes were treated overnight with anti-JAK2, anti-p-JAK2, anti-STAT3, and anti-p-STAT3 antibodies (1 : 1,000) at 4°C. Subsequently, the membranes were treated with a goat anti-rabbit IgG-HRP (1 : 5,000) secondary antibody for 1 hr at room temperature and then with ECL in a dark room for developing color. The Bio-Rad full-function imaging system was used for image acquisition, and Image-ProPlus was used for analyzing optical density. *β*-Actin was used as an internal reference to calculate the relative expression of each protein.

### 2.9. Statistical Analysis

Data were analyzed using SPSS 20.0 statistical software (SPSS Inc., Chicago, IL, USA) and expressed as mean ± standard deviation (*X* ± *S*). One-way analysis of variance (ANOVA) was used for comparing data from multiple groups. *P* < 0.05 indicated that the differences in data were statistically significant.

## 3. Results

### 3.1. Effect of Morroniside Treatment on Intraocular Inflammation and Histopathology in EIU Mice

**Figure 1:**
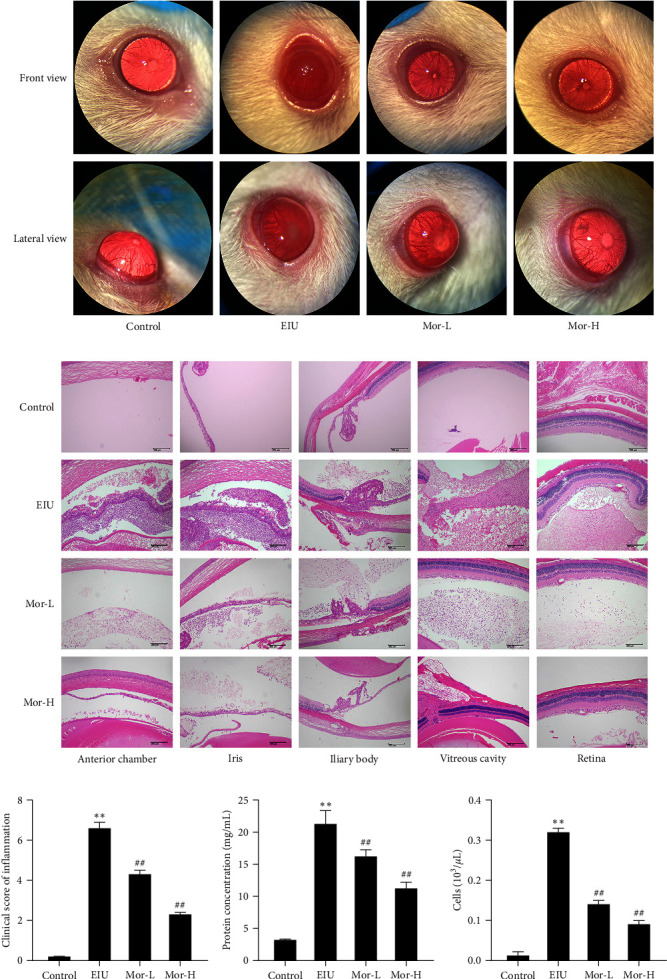
Effect of morroniside on intraocular inflammation and histopathology in EIU mice. (a) Inflammation in each group under slit lamp microscopy at 24 hr after LPS injection (front and lateral views). (b) Observation of HE staining patterns in the anterior chamber, iris, ciliary body, vitreous cavity, and retinal tissues (100×, scale bar, 200 *μ*m). (c) The clinical score in each group at 24 hr after LPS challenge. (d) The number of inflammatory cells in the aqueous humor was counted after 24 hr. (e) The protein concentrations in the aqueous humor were measured after 24 hr. Mean ± SD from three independent samples are shown. Comparison with the control group:  ^*∗∗*^*P* < 0.01. Comparison with the EIU group: ^#^*P* < 0.05, ^##^*P* < 0.01.

Compared with those in the control group, both the frontal and lateral views in the EIU group showed an inflammatory response, including congestion and fibrinous membrane formation. However, this effect was mitigated in the low- and high-dose Mor treatment groups (Figures 1(a) and 1(c)). To characterize the morphological changes occurring in uveitis, the HE staining patterns in the anterior chamber, iris, ciliary body, vitreous cavity, and retinal tissues were observed. In contrast to that in the control group, some inflammatory cell infiltrates were observed in the anterior chamber, epithelial and connective tissue layers of ciliary tissues, posterior layer of the iris tissue, vitreous tissue, and retina in the EIU group. The abovementioned adverse effects improved significantly in the Mor-H group (Figure 1(b)). Moreover, the number of inflammatory cells and the protein exudate level in the aqueous humor were significantly higher in the EIU group. Meanwhile, low-dose and high-dose Mor treatments significantly reduced the number of inflammatory cells (Figures 1(d) and 1(e)). These findings indicated that Mor relieves intraocular inflammation and histopathological changes.

### 3.2. Effect of Morroniside on the Level of Inflammatory Factors and Inflammatory Cell Counts in EIU Mice

**Figure 2:**
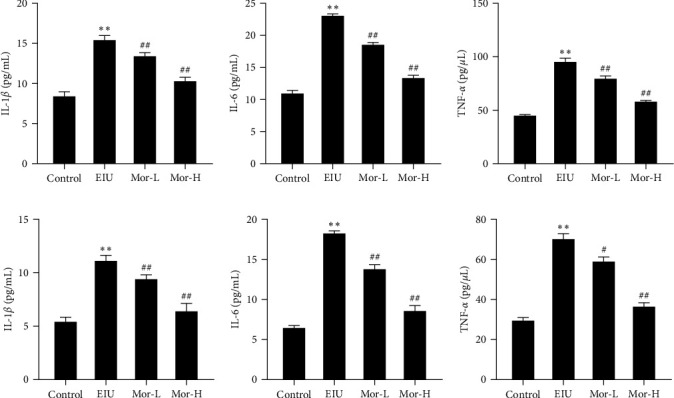
Effect of morroniside on the levels of inflammatory factors and inflammatory cell counts in EIU mice. (a) The levels of IL-1*β*, IL-6, and TNF-*α* in the ciliary body. (b) The levels of IL-1*β*, IL-6, and TNF-*α* in the retinal cells. Mean ± SD from three independent samples are shown. Comparison with the control group:  ^*∗∗*^*P* < 0.01. Comparison with the EIU group: ^#^*P* < 0.05, ^##^*P* < 0.01.

To further verify the effect of morroniside on the level of inflammatory factors in EIU mice, relevant parameters were investigated using ELISA. Compared with those in the control group, the levels of IL-1*β*, IL-6, and TNF-*α* were significantly higher in the iris ciliary and retinal cells of mice in the EIU group (*P* < 0.01). Compared with those in the EIU group, the IL-1*β*, IL-6, and TNF-*α* levels in the iris ciliary and retinal cells of mice in the Mor-L and Mor-H groups were significantly lower (*P* < 0.05). This indicated the ameliorating effects of Mor on the inflammatory response induced by EIU (Figure 2).

### 3.3. Effect of Morroniside on M1/M2 Polarization in Iris Ciliary Body Macrophages in EIU Mice

**Figure 3:**
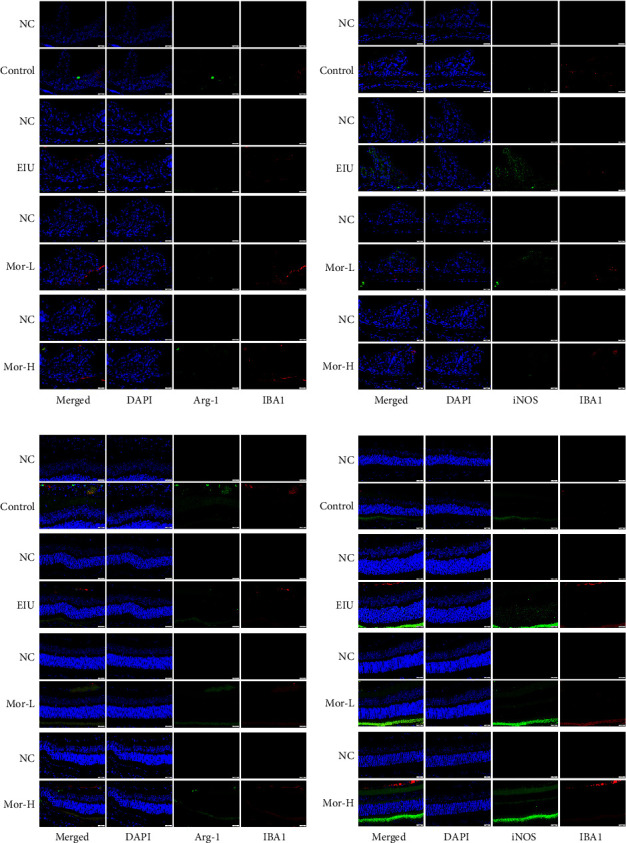
Effect of morroniside on M1/M2 polarization in iris ciliary body macrophages in EIU mice. (a) Arg-1 expression in the ciliary body tissues of mice (40×, scale bar, 20 *μ*m). (b) iNOS expression in the ciliary body tissues of mice (40×, scale bar, 20 *μ*m). (c) Expression of Arg-1 in the retinal tissues of mice (40×, scale bar, 20 *μ*m). (d) Expression of iNOS in the retinal tissues of mice (40×, scale bar, 20 *μ*m).

We next measured Arg-1 and iNOS expression in the ciliary body and retinal tissues. As shown in Figure 3, the expression of Arg-1 and iNOS corresponded with a green fluorescence. Arg-1 expression decreased and iNOS expression increased in the ciliary body and retinal tissues of mice from the EIU group, whereas Arg-1 expression increased and iNOS expression decreased in the ciliary body and retinal tissues of mice from the Mor-L and Mor-H groups. Thus, morroniside modulated macrophage polarization in EIU and caused macrophage polarization toward the M2 phenotype with anti-inflammatory effects.

### 3.4. Effect of Morroniside on the JAK/STAT Signaling Pathway in EIU Mice

**Figure 4:**
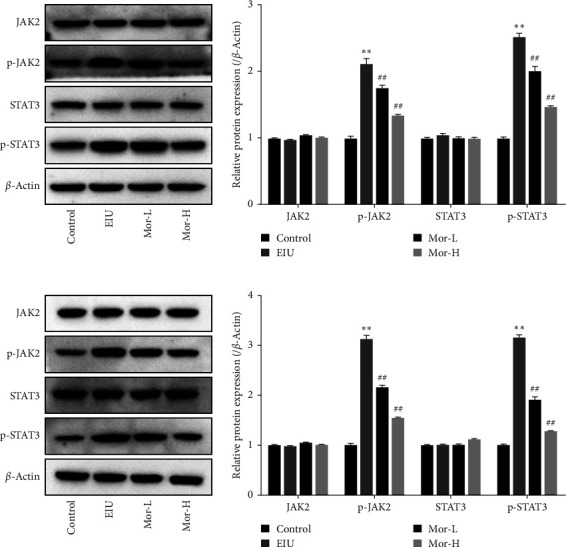
Effect of morroniside on the JAK/STAT signaling pathway in EIU mice. (a) JAK2, p-JAK2, STAT3, and p-STAT3 protein expression in ciliary body tissues. (b) JAK2, p-JAK2, STAT3, and p-STAT3 protein expression in retinal tissues. Means ± SD from three independent samples are shown. The data in western blot assays were expressed after normalization to *β*-actin expression. Comparison with the control group:  ^*∗∗*^*P* < 0.01. Comparison with the EIU group: ^##^*P* < 0.01.

Since the JAK/STAT pathway may contribute to the regulation of macrophage polarization, we speculated that morroniside may affect macrophage polarization by regulating the JAK/STAT pathway. We analyzed the expression of JAK2, p-JAK2, STAT3, and p-STAT3 proteins in the ciliary body and retinal tissues using western blotting. p-JAK2 and p-STAT3 expression in the ciliary body and retina in the EIU group increased compared with that in the control group (*P* < 0.01), whereas p-JAK2 and p-STAT3 expression decreased in the Mor-L and Mor-H groups compared with that in the EIU group (*P* < 0.01, Figure 4). These data indicated that Mor inhibits the activation of the JAK/STAT signaling pathway.

### 3.5. Morroniside-Mediated JAK/STAT Signaling Affects EIU Mice

**Figure 5:**
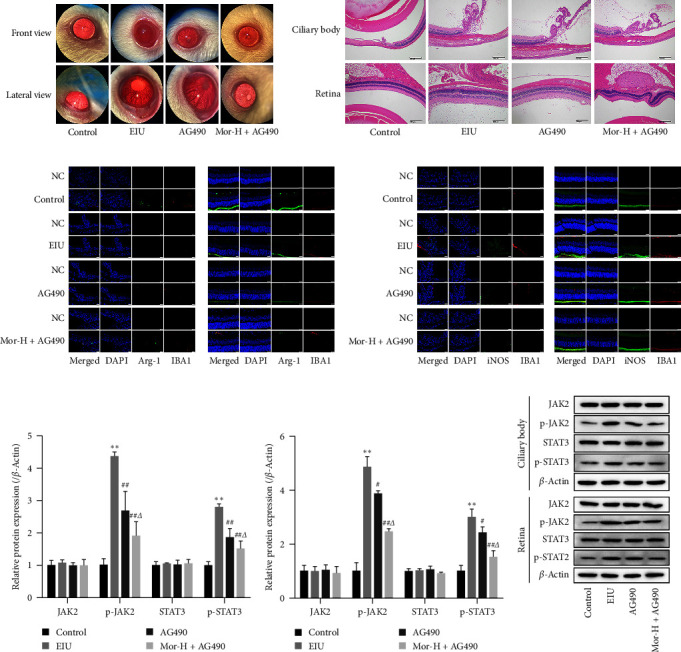
Morroniside treatment affects the JAK/STAT signaling pathway in EIU mice. (a) Inflammation in each group observed under slit lamp microscopy (frontal and lateral views). (b) Observation of hematoxylin–eosin staining in ciliary body and retinal tissues (100×, scale bar, 200 *μ*m). (c) Expression of Arg-1 in the ciliary body and retinal tissues of mice (40×, scale bar, 20 *μ*m). (d) Expression of iNOS in the ciliary body and retinal tissues of mice (40×, scale bar, 20 *μ*m). (e, g) Protein expression of JAK2, p-JAK2, STAT3, and p-STAT3 in ciliary body tissues. (f, g) Protein expression of JAK2, p-JAK2, STAT3, and p-STAT3 in retinal tissues. Means ± SD from three independent samples are shown. Data in western blot assays were expressed after normalization to *β*-actin levels. Comparison with the control group:  ^*∗∗*^*P* < 0.01; comparison with the EIU group: ^#^*P* < 0.05, ^##^*P* < 0.01; comparison with the AG490 group: ^*Δ*^*P* < 0.05.

To confirm the protective effect of morroniside in EIU mediated via the inhibition of the JAK/STAT pathway, we further treated EIU mice using a JAK inhibitor (AG490) or Mor-H + AG490. Compared with those in the AG490 group, the frontal and lateral inflammatory manifestations in the slit lamp microscopy experiment significantly improved in the Mor-H + AG490 group (Figure 5(a)). Additionally, histopathological damage in the ciliary body and retinal tissues was significantly attenuated in the Mor-H+AG490 group (Figure 5(b)). In addition, compared with that in the AG490 group, Arg-1 expression was increased and iNOS expression was reduced in ciliary and retinal tissues in the Mor-H + AG490 group (Figures 5(c) and 5(d)). The results of western blot analysis showed that p-JAK2 and p-STAT3 protein expression was significantly lower in the ciliary body and retinal tissues of the Mor-H + AG490 group compared with that in the AG490 group (*P* < 0.05, Figures 5(e)–5(g)). These results demonstrated that morroniside exerts a protective effect on EIU by promoting M2 polarization by suppressing the JAK/STAT pathway.

## 4. Discussion

In the present study, we observed that morroniside alleviated the effects of EIU, and its protective effect occurred at least partly through macrophage polarization for inhibiting the inflammatory response. Furthermore, morroniside may affect macrophage polarization by inhibiting the activation of the JAK/STAT signaling pathway, which helps alleviate the effects of EIU.

Inflammation plays a vital role in EIU, and the goal of EIU treatment is to reduce inflammation and prevent damage to ocular tissues [20]. To date, various corticosteroid regimens have been the mainstay of EIU treatment; however, the uncontrollable side effects of these treatments can cause extensive tissue damage, the most common ocular side effects being glaucoma, cataract, ptosis, and mydriasis [21, 22]. In the present study, treatment with morroniside significantly alleviated the inflammatory response in EIU mice. The aqueous humor cell count is a useful indicator of the integrity of the blood-aqueous humor barrier in the EIU model. LPS treatment elevated the aqueous humor cell count, consistent with evidence from previous studies on uveitis [23, 24]. LPS treatment also increased the concentrations of IL-1*β*, IL-6, and TNF-*α* in the iris ciliary and retinal cells of EIU mice, whereas the concentrations of these three inflammatory factors and the aqueous humor cell count were significantly lower in the Mor group, which was consistent with the inhibition of inflammatory factors by Mor. Mor also significantly reversed the histopathological changes in EIU mice, suggesting that the anti-inflammatory effect of Mor on LPS stimulation was dependent on the inhibition of IL-1*β*, IL-6, and TNF-*α* expression, which helped alleviate the effects of EIU.

Macrophages are important immune cells involved in humoral and cellular immunity, and M1 and M2 macrophages, as two subpopulations of macrophages exhibiting different effects in inflammation, are considered to play a crucial role in various inflammatory diseases. Macrophage polarization is also considered important for regulating the progression of inflammation [25, 26]. Some studies have reported that the differentiation of M1 macrophages upon LPS stimulation leads to the release of proinflammatory factors such as IL-6 and TNF-*α* and the increased expression of inducible nitric oxide synthase (iNOS), which promotes inflammation [27]. Stimulated by IL-4 and IL-13 signaling, M2 macrophage surface markers arginase-1 (Arg-1) and CD206 show high expression, which has anti-inflammatory and pro-tissue repair effects [28]. In this study, iNOS expression was significantly elevated in the ciliary body and retinal tissues of EIU mice, suggesting that macrophages in the ciliary body and retina tended to exhibit M1 polarization, disrupting the proinflammatory/anti-inflammatory balance. After the EIU mice were treated with morroniside, Arg-1 expression in the ciliary body and retinal tissues increased significantly, indicating the trend toward M2 macrophage polarization in the ciliary body and retina. In addition, higher doses of Mor were more effective, indicating that at higher doses, Mor may exert a more pronounced effect on EIU treatment.

The regulation of macrophage polarization and function involves a combination of factors, including the regulation of multiple signaling pathways and transduction and transcriptional networks [29]. Among them, the most common is the JAK2/STAT3 pathway. JAK2 is a protein tyrosine kinase that is present in the cytoplasm and regulates the expression of genes encoding downstream STATs. At this level, JAK2 can directly influence the phosphorylation level of STAT3, thus affecting cell proliferation, migration, and invasion [30]. The JAK2/STAT3 pathway has been shown to induce the polarization of macrophages to the M1 phenotype, thus inducing the synthesis and secretion of proinflammatory factors at high levels [31]. In the present study, Mor did not exert a significant effect on the protein expression of JAK2 and STAT3 in EIU mice at low or high concentrations, but it significantly downregulated protein phosphorylation. This suggested that the JAK2/STAT3 pathway was inhibited by Mor in EIU mice. Consistent with the detection of macrophage polarization, the inhibition of phosphorylation in the JAK2/STAT3 pathway was more pronounced upon treatment with high-dose Mor. Further treatment with JAK inhibitors revealed that Mor reduced the tendency of macrophages to polarize to the M1 phenotype by inhibiting the JAK2/STAT3 pathway, which in turn exerted a protective effect on EIU.

In summary, Mor inhibits EIU development by suppressing phosphorylation in the JAK2/STAT3 signaling pathway and promoting macrophage polarization to the M2 phenotype, thereby downregulating the expression of proinflammatory factors and upregulating the expression of anti-inflammatory factors. However, in this study, we only preliminarily investigated the regulatory effect of Mor on macrophage polarization in EIU treatment. More comprehensive experimental validation is necessary to explore the mechanism underlying EIU treatment using Mor.

## Figures and Tables

**Table 1:** The grading system used for ocular inflammation.

Clinical signs	Grade of uveitis (score)
Iris hyperemia	
Absent	0
Mild	1
Moderate	2
Severe	3
Pupil	
Normal	0
Miosis	1
Exudate in anterior chamber	
Absent	0
Small	1
Large	2
Hypopyon	
Absent	0
Present	1
Maximum possible score	7

## Data Availability

The data used to support the findings of this study are included in the article.
